# Chronic rhinosinusitis and premorbid autoimmune diseases: a population-based case–control study

**DOI:** 10.1038/s41598-020-75815-x

**Published:** 2020-10-29

**Authors:** Liang-Chun Shih, Hua-Hsin Hsieh, Gregory J. Tsay, Ivan T. Lee, Yung-An Tsou, Cheng-Li Lin, Te-Chun Shen, Da-Tian Bau, Chih-Jaan Tai, Chia-Der Lin, Ming-Hsui Tsai

**Affiliations:** 1 Department of Otorhinolaryngology, China Medical University Hospital, Taichung, 40447 Taiwan; 2 Graduate Institute of Biomedical Sciences, China Medical University, Taichung, 40447 Taiwan; 3 Terry Fox Cancer Research Laboratory, Department of Medical Research, China Medical University Hospital, Taichung, 40447 Taiwan; 4 Division of Immunology and Rheumatology, Department of Internal Medicine, China Medical University Hospital, Taichung, 40447 Taiwan; 5 Division of Allergy, Immunology, and Rheumatology, Department of Pediatrics, Stanford University School of Medicine, Stanford, CA 94305 USA; 6 School of Medicine, China Medical University, Taichung, 40447 Taiwan; 7 Management Office for Health Data, China Medical University Hospital, Taichung, 40447 Taiwan; 8 Division of Pulmonary and Critical Care Medicine, Department of Internal Medicine, China Medical University Hospital, No. 2, Yude Rode, Taichung, 40447 Taiwan

**Keywords:** Diseases, Health care, Rheumatology, Risk factors

## Abstract

Evidence shows that chronic rhinosinusitis (CRS) is associated with prior presence of autoimmune diseases; however, large-scale population-based studies in the literature are limited. We conducted a population-based case–control study investigating the association between CRS and premorbid autoimmune diseases by using the National Health Insurance Research Database in Taiwan. The CRS group included adult patients newly diagnosed with CRS between 2001 and 2013. The date of diagnosis was defined as the index date. The comparison group included individuals without CRS, with 1:4 frequency matching for gender, age, and index year. Premorbid diseases were forward traced to 1996. Univariate and multivariate logistic regression was performed to estimate odds ratios (ORs) and 95% confidence intervals. The CRS group consisted of 30,611 patients, and the comparison group consisted of 122,444 individuals. Patients with CRS had a higher significant association with premorbid autoimmune diseases (adjusted OR 1.39 [1.28–1.50]). Specifically, patients with CRS had a higher significant association with ankylosing spondylitis, polymyositis, psoriasis, rheumatoid arthritis, sicca syndrome, and systemic lupus erythematosus (adjusted OR 1.49 [1.34–1.67], 3.47 [1.12–10.8], 1.22 [1.04–1.43], 1.60 [1.31–1.96], 2.10 [1.63–2.72], and 1.69 [1.26–2.25]). In subgroup analysis, CRS with and without nasal polyps demonstrated a significant association with premorbid autoimmune diseases (adjusted OR 1.34 [1.14–1.58] and 1.50 [1.38–1.62]). In addition, CRS with fungal and non-fungal infections also demonstrated a significant association with premorbid autoimmune diseases (adjusted OR 2.02 [1.72–2.49] and 1.39 [1.28–1.51]). In conclusion, a significant association between CRS and premorbid autoimmune diseases has been identified. These underlying mechanisms need further investigation.

## Introduction

Chronic rhinosinusitis (CRS) is characterized by inflammation of the sinus mucosa, which leads to clinical symptoms such as nasal obstruction, mucus accumulation, and anosmia^1,2^. CRS is a heterogeneous disease with multiple presentations, and it is commonly subcategorized as CRS with nasal polyps (CRSwNP) and CRS without nasal polyps (CRSsNP)^3,4^. Specifically, CRSwNP is characterized by T-help 2 responses and eosinophilic inflammation, and CRSsNP is characterized by T-help 1 responses and neutrophilic inflammation^5^. Several subtypes, such as chronic bacterial and fungal infections, associated with different immune pathways or presentations are also proposed^6,7^. Clinicians should understand the underlying inflammatory mechanism to achieve favorable treatment.

Prior studies on the association between CRS and autoimmune diseases have been carried out, but they are limited^8−11^. Chandra et al.^12^ conducted a cross-sectional study and reported the prevalence of several autoimmune diseases, including psoriasis (0.20%, 4009/1,970,695), lupus (0.14%, 2788/1,970,695), rheumatoid arthritis (0.14%, 2721/1,970,695), multiple sclerosis (0.08%, 1533/1,970,695), and ankylosing spondylitis (0.04%, 777/1,970,695). They further reported the prevalence of CRS in these autoimmune diseases: ankylosing spondylitis (6.05%, 47/777), rheumatoid arthritis (5.0%, 136/2721), lupus (3.9%, 108/2788), psoriasis (3.8%, 152/4009), and multiple sclerosis (1.4%, 21/1533). They also identified the proportion of CRSwNP (*n* = 43, 9.3%) and CRSsNP (*n* = 421, 91.7%) among these autoimmune diseases: ankylosing spondylitis [0.13% (1/777) and 5.9% (46/777)], rheumatoid arthritis [0.44% (12/2721) and 4.6% (124/2721)], lupus [0.29% (8/2788) and 3.6% (100/2788)], psoriasis [0.47% (19/4009) and 3.3% (133/4009)], and multiple sclerosis [0.2% (3/1533) and 1.2% (18/1533)]. Another cross-sectional study investigated various comorbid diseases in CRS cases (n = 5734) and controls (n = 17,202), which reported an increased prevalence of several autoimmune diseases in patients with CRS compared to controls as follows: RA (7.2 vs. 4.0%), AS (1.5 vs. 1.1%) and SLE (0.3% vs. 0.2%). The authors concluded that CRS is significantly associated with rheumatoid arthritis [adjusted odds ratio (adjusted OR) = 1.85, 95% confidence interval (CI) = 1.63–2.10] and ankylosing spondylitis (adjusted OR 1.39, 95% CI 1.07–1.81), but not with systemic lupus erythematosus (adjusted OR 1.50, 95% CI 0.85–2.66)^13^.

The association between CRS and premorbid autoimmune diseases is an essential issue. First, CRS is a multifactorial disease and has a broad spectrum of associations, ranging from genetics to comorbid diseases and environmental factors. Autoimmune diseases have been considered a risk factor for CRS^9^. Second, patients with autoimmune diseases may share the same or similar pathogenic mechanisms for developing CRS or its certain subtypes. Third, autoimmune diseases have been considered a predictive factor for frequent acute CRS exacerbations^14^. Autoimmune diseases may contribute to poor CRS prognosis. To date, the association between CRS (or its subtypes) and premorbid autoimmune diseases remains largely uncertain. Therefore, we aimed to use the National Health Insurance Research Database (NHIRD) in Taiwan and to conduct a retrospective case–control study to clarify whether CRS and its subtypes are associated with premorbid autoimmune diseases.

## Materials and methods

### Data sources

This is a retrospective study utilizing data from Longitudinal Health Insurance Database 2000 (LHID2000), which is a subset of the NHIRD. The LHID2000 included 1,000,000 persons recruited in 2000 and contained their information of demographics, records of outpatient visit and hospitalizations, prescription details (prescribed medications, dosages, and expenditure amounts), and diagnosed diseases (coded in accordance with the International Classification of Diseases, 9th Revision, Clinical Modification, ICD-9-CM) from 1996 to 2013. This study was granted the approval by the Research Ethics Committee of the China Medical University and Hospital (CMUH-104-REC2-115). All methods were performed in accordance with the Strengthening the Reporting of Observational studies in Epidemiology (STROBE) guideline. Informed consent was unnecessary for the de-identified data and waived by Research Ethics Committee of the China Medical University and Hospital.

### Study population

The CRS group included adult patients who were newly diagnosed with CRS between 2001 and 2013. The date of diagnosis was defined as the index date. The diagnostic criteria of CRS (ICD-9-CM 473) were more than two outpatient records or at least one hospitalization record, and all patients were diagnosed by otorhinolaryngology physicians^15^. CRSwNP was defined as patients who were both coded with CRS (ICD-9-CM 473) and nasal polyps (ICD-9-CM 471). CRSsNP was defined as patients with CRS other than CRSwNP. CRS with fungal infection was defined as patients who were both coded with CRS (ICD-9-CM 473) and fungal infection (ICD-9-CM 110-118). CRS with non-fungal infection was defined as patients with CRS other than fungal infection. The comparison group included individuals without CRS, with 1:4 frequency matching for gender, age, and index year.

### Study outcome and comorbidities

The primary interests of the study were the presence of autoimmune diseases, including ankylosing spondylitis (ICD-9-CM 720.0), Behçet’s disease (ICD-9-CM 136.1), dermatomyositis (ICD-9-CM 710.3), multiple sclerosis (ICD-9-CM 340), polymyositis (ICD-9-CM 710.4), psoriasis (ICD-9-CM 696.0, 696.1), rheumatoid arthritis (ICD-9-CM 714.0), sicca syndrome (ICD-9-CM 710.2), systemic lupus erythematosus (ICD-9-CM 710.0), and systemic sclerosis (ICD-9-CM 710.1)^16,17^. In addition, we evaluated the presence of several comorbidities to investigate their relation to CRS. These comorbidities included allergic rhinitis (ICD-9-CM 477), asthma (ICD-9-CM 493), chronic obstructive pulmonary disease (ICD-9-CM 496), depression (ICD-9-CM 296.2, 296.3, 300.4, 311), diabetes mellitus (ICD-9-CM 250), gastroesophageal reflux disease (ICD-9-CM 530.11, 530.81), hyperlipidemia (ICD-9-CM 272), hypertension (ICD-9-CM 401), ischemic heart disease (ICD-9-CM 410-414), and obesity (ICD-9-CM 278.0). These diagnoses had to meet also more than two outpatient records or at least one hospitalization record before the index date and could be traced back to 1996.

### Statistical analyses

The baseline characteristics were shown as frequency count and percent, and chi-squared test was applied to compare the differences in gender, age, monthly income, and urbanization among the CRS and comparison groups. Univariate and multivariate logistic regression was adopted for risk assessment, and the results were presented using ORs and 95% CIs. For the multivariate logistic regression model, the adjusted variables included gender, age, monthly income, urbanization, and comorbidities. Significance levels were defined as two-sided *p* < 0.05. All analyses were carried out with SAS 9.4 software (SAS Institute, Cary, NC, USA).

## Results

The CRS group consisted of 30,611 patients, and the comparison group consisted of 122,444 individuals (Table 1). The distributions of gender, age, and urbanization did not differ between the CRS group and the comparison group. Patients with CRS had a higher monthly income than individuals without CRS. As for gender, 51.7% of patients with CRS were women. As for age, 46.3% of patients with CRS were 40–64 years old, 40.1% of patients with CRS were 20–39 years old, and only 13.6% of patients with CRS were more than 65 years old.

**Table 1:** Demographic characteristics of individuals with and without chronic rhinosinusitis.

Variables	Non-CRS		CRS		*p* value
	N = 122,444		N = 30,611		
	n	%	n	%	
**Sex**					0.99
Women	63,256	51.7	15,814	51.7	
Men	59,188	48.3	14,797	48.3	
**Age**					0.82
20–39	49,351	40.3	12,280	40.1	
40–64	56,473	46.1	14,176	46.3	
≥ 65	16,620	13.6	4155	13.6	
**Monthly income**^a^					< 0.001
≤ 15,840	46,526	38.0	11,177	36.5	
15,841–24,999	34,835	28.5	8434	27.6	
≥ 25,000	41,083	33.6	11,000	35.9	
**Urbanization**					0.33
City	74,798	61.1	18,606	60.8	
Rural area	47,646	38.9	12,005	39.2	

^a^1 New Taiwan dollar equals to 0.03 US dollar.

Patients with CRS were significantly more likely to have various premorbid diseases (Table 2), including autoimmune diseases (adjusted OR 1.39, 95% CI 1.28–1.50), allergic rhinitis (adjusted OR 4.38, 95% CI 4.26–4.51), asthma (adjusted OR 2.14, 95% CI 1.89–2.39), chronic obstructive pulmonary disease (adjusted OR 1.71, 95% CI 1.65–1.78), depression (adjusted OR 1.21, 95% CI 1.15–1.28), gastroesophageal reflux disease (adjusted OR 1.45, 95% CI 1.35–1.56), hyperlipidemia (adjusted OR 1.13, 95% CI 1.08–1.17), hypertension (adjusted OR 1.05, 95% CI 1.01–1.10), ischemic heart disease (adjusted OR 1.27, 95% CI 1.21–1.33), and obesity (adjusted OR 1.18, 95% CI 1.07–1.31).

**Table 2:** Univariate and multivariate logistic regression for premorbid diseases between CRS group and non-CRS group.

Variables	Non-CRS n (%)	CRS n (%)	Odds ratio	
			Crude (95% CI)	Adjusted^a^ (95% CI)
Autoimmune diseases	2404 (1. 96)	995 (3.25)	1.68 (1.56–1.81)***	1.39 (1.28–1.50)***
Allergic rhinitis	19,556 (16.0)	15,124 (49.4)	5.14 (5.00–5.28)***	4.38 (4.26–4.51)***
Asthma	8069 (6.59)	4491 (14.7)	2.44 (2.34–2.53)***	2.14 (1.89–2.39)***
COPD	21,658 (17.7)	10,638 (34.8)	2.48 (2.41–2.55)***	1.71 (1.65–1.78)***
Depression	5984 (4.89)	2511 (8.20)	1.74 (1.66–1.83)***	1.21 (1.15–1.28)***
Diabetes	6143 (5.02)	1684 (5.50)	1.10 (1.04–1.17)***	1.04 (0.93–1.11)
GERD	2524 (2.06)	1547 (5.05)	2.53 (2.37–2.70)***	1.45 (1.35–1.56)***
Hyperlipidemia	19,468 (15.9)	6576 (21.5)	1.45 (1.40–1.49)***	1.13 (1.08–1.17)***
Hypertension	28,315 (23.1)	8300 (27.1)	1.24 (1.20–1.27)***	1.05 (1.01–1.10) *
IHD	14,432 (11.8)	5262 (17.2)	1.55 (1.50–1.61)***	1.27 (1.21–1.33)***
Obesity	1532 (1.25)	577 (1.88)	1.52 (1.38–1.67)***	1.18 (1.07–1.31)***

*CI* confidence interval, *COPD* chronic obstructive pulmonary disease, *CRS* chronic rhinosinusitis, *GERD* gastroesophageal reflux disease, *IHD* ischemic heart disease.

*p < 0.05, ***p < 0.001.

^a^Adjusted for gender, age, monthly income, urbanization, and comorbidities.

Among the selected autoimmune diseases in Table 3, patients with CRS had a higher significant association to have ankylosing spondylitis (adjusted OR 1.49, 95% CI 1.34–1.67), polymyositis (adjusted OR 3.47, 95% CI 1.12–10.8), psoriasis (adjusted OR 1.22, 95% CI 1.04–1.43), rheumatoid arthritis (adjusted OR 1.60, 95% CI 1.31–1.96), sicca syndrome (adjusted OR 2.10, 95% CI 1.63–2.72), and systemic lupus erythematosus (adjusted OR 1.69, 95% CI 1.26–2.25).

**Table 3:** Univariate and multivariate logistic regression for premorbid autoimmune diseases between CRS group and non-CRS group.

Variables	Non-CRS n (%)	CRS n (%)	Odds ratio	
			Crude (95% CI)	Adjusted^a^ (95% CI)
Autoimmune diseases	2404 (1.96)	995 (3.25)	1.68 (1.56–1.81)***	1.39 (1.28–1.50)***
Ankylosing spondylitis	1109 (0.91)	468 (1.53)	1.70 (1.53–1.90)***	1.49 (1.34–1.67)***
Behçet’s disease	12 (0.01)	7 (0.02)	2.43 (0.92–3.95)	2.35 (0.90–6.15)
Dermatomyositis	9 (0.01)	1 (0.00)	0.45 (0.06–3.52)	0.52 (0.07–4.12)
Multiple sclerosis	0 (0.00)	0 (0.00)	–	–
Polymyositis	6 (0.00)	7 (0.02)	4.67 (1.57–13.9)**	3.47 (1.12–10.8)*
Psoriasis	649 (0.53)	224 (0.73)	1.38 (1.19–1.61)***	1.22 (1.04–1.43)*
Rheumatoid arthritis	336 (0.27)	146 (0.48)	1.74 (1.44–2.12)***	1.60 (1.31–1.96)***
Sicca syndrome	155 (0.13)	105 (0.34)	2.72 (2.12–3.48)***	2.10 (1.63–2.72)***
SLE	147 (0.12)	74 (0.24)	2.02 (1.53–2.67)***	1.69 (1.26–2.25)***
Systemic sclerosis	29 (0.02)	5 (0.02)	0.69 (0.27–1.78)	0.64 (0.24–1.69)

*CI* confidence interval, *CRS* chronic rhinosinusitis, *SLE* systemic lupus erythematosus.

*p < 0.05, **p < 0.01, ***p < 0.001.

^a^Adjusted for gender, age, monthly income, urbanization, and comorbidities.

Furthermore, we classified CRS into CRSsNP (n = 24,907, 81.4%) and CRSwNP (n = 5704, 18.6%; Table 4). Patients with CRSsNP had a higher significant association to have overall autoimmune diseases (crude OR 1.73, 95% CI 1.54–1.94; adjusted OR 1.50, 95% CI 1.38–1.62), specifically for ankylosing spondylitis (adjusted OR 1.50, 95% CI 1.33–1.70), polymyositis (adjusted OR 3.54, 95% CI 1.09–11.6), psoriasis (adjusted OR 1.29, 95% CI 1.09–1.52), rheumatoid arthritis (adjusted OR 1.51, 95% CI 1.22–1.88), sicca syndrome (adjusted OR 2.31, 95% CI 1.77–3.01), and systemic lupus erythematosus (adjusted OR 1.83, 95% CI 1.36–2.47). Patients with CRSwNP also had a higher significant association to have overall autoimmune diseases (crude OR 1.44, 95% CI 1.23–1.70; adjusted OR 1.34, 95% CI 1.14–1.58), specifically for ankylosing spondylitis (adjusted OR 1.46, 95% CI 1.16–1.83) and rheumatoid arthritis (adjusted OR 2.03, 95% CI 1.40–2.94). Table 5 demonstrates logistic regression for premorbid autoimmune diseases between CRSwNP and CRSsNP. CRSwNP had less association with sicca syndrome (adjusted OR 0.45, 95% CI 0.22–0.92) than CRSsNP.

**Table 4:** Univariate and multivariate logistic regression for premorbid autoimmune diseases between CRS subgroups (CRSwNP and CRSsNP) and non-CRS group.

Variables	CRSsNP	CRSwNP	Odds ratio (CRSsNP vs. non-CRS)		Odds ratio (CRSwNP vs. non-CRS)	
	N = 24,907 n (%)	N = 5704 n (%)	Crude (95% CI)	Adjusted^a^ (95% CI)	Crude (95% CI)	Adjusted^a^ (95% CI)
Autoimmune diseases	835 (3.35)	160 (2.81)	1.73 (1.54–1.94)***	1.50 (1.38–1.62)***	1.44 (1.23–1.70)***	1.34 (1.14–1.58)***
Ankylosing spondylitis	386 (1.55)	82 (1.44)	1.73 (1.54–1.94)***	1.50 (1.33–1.70)***	1.60 (1.27–2.00)***	1.46 (1.16–1.83)**
Behçet’s disease	5 (0.02)	2 (0.04)	2.05 (0.72–5.82)	2.03 (0.69–5.99)	3.61 (0.81–16.1)	3.66 (0.82–16.4)
Dermatomyositis	1 (0.00)	0 (0.00)	0.55 (0.07–4.31)	0.65 (0.08–5.17)	–	–
Multiple sclerosis	0 (0.00)	0 (0.00)	–	–	–	–
Polymyositis	6 (0.02)	1 (0.02)	4.92 (1.59–15.2)**	3.54 (1.09–11.6)*	3.58 (0.43–29.7)	3.12 (0.37–26.3)
Psoriasis	193 (0.77)	31 (0.54)	1.47 (1.25–1.72)***	1.29 (1.09–1.52)**	1.03 (0.72–1.47)	0.93 (0.65–1.33)
Rheumatoid arthritis	115 (0.46)	31 (0.54)	1.69 (1.36–2.08)***	1.51 (1.22–1.88)***	1.99 (1.37–2.87)***	2.03 (1.40–2.94)***
Sicca syndrome	97 (0.39)	8 (0.14)	3.09 (2.39–3.98)***	2.31 (1.77–3.01)***	1.11 (0.54–2.26)	1.03 (0.51–2.11)
SLE	67 (0.27)	7 (0.12)	2.24 (1.68–3.00)***	1.83 (1.36–2.47)***	1.02 (0.48–2.18)	0.98 (0.46–2.10)
Systemic sclerosis	4 (0.02)	1 (0.02)	0.68 (0.24–1.93)	0.62 (0.21–1.80)	0.74 (0.10–5.44)	0.74 (0.10–5.48)

*CI* confidence interval, *CRS* chronic rhinosinusitis, *CRSsNP* chronic rhinosinusitis without nasal polyp, *CRSwNP* chronic rhinosinusitis with nasal polyp, *SLE* systemic lupus erythematosus.

*p < 0.05, **p < 0.01, ***p < 0.001.

^a^Adjusted for gender, age, monthly income, urbanization, and comorbidities.

**Table 5:** Univariate and multivariate logistic regression for premorbid autoimmune diseases between CRSwNP and CRSsNP.

Variables	Odds ratio (CRSwNP vs. CRSsNP)	
	Crude (95% CI)	Adjusted^a^ (95% CI)
Autoimmune diseases	0.83 (0.70–0.99)*	0.89 (0.75–1.06)
Ankylosing spondylitis	0.93 (0.73–1.18)	0.97 (0.76–1.23)
Behçet’s disease	1.75 (0.34–9.02)	1.63 (0.31–8.48)
Dermatomyositis	–	–
Multiple sclerosis	–	–
Polymyositis	0.73 (0.09–6.05)	0.85 (0.10–7.14)
Psoriasis	0.70 (0.48–1.02)	0.70 (0.48–1.03)
Rheumatoid arthritis	1.18 (0.79–1.75)	1.36 (0.91–2.03)
Sicca syndrome	0.36 (0.18–0.74)**	0.45 (0.22–0.92)**
SLE	0.46 (0.21–0.99)*	0.53 (0.24–1.15)
Systemic sclerosis	1.09 (0.12–7.8)	1.35 (0.15–12.2)

*CI* confidence interval, *CRSsNP* chronic rhinosinusitis without nasal polyp, *CRSwNP* chronic rhinosinusitis with nasal polyp, *SLE* systemic lupus erythematosus.

*p < 0.05, **p < 0.01.

^a^Adjusted for gender, age, monthly income, urbanization, and comorbidities.

CRS was thus classified into non-fungal infection (*n* = 26,778, 87.5%) and fungal infection (*n* = 3833, 12.5%; Table 6). CRS with non-fungal infection had a higher significant association with overall autoimmune diseases (crude OR 1.56, 95% CI 1.44–1.70; adjusted OR 1.39, 95% CI 1.28–1.51), specifically for ankylosing spondylitis (adjusted OR 1.44, 95% CI 1.28–1.62), polymyositis (adjusted OR 3.45, 95% CI 1.07–11.1), rheumatoid arthritis (adjusted OR 1.59, 95% CI 1.29–1.96), sicca syndrome (adjusted OR 2.10, 95% CI 1.61–2.75), and systemic lupus erythematosus (adjusted OR 1.45, 95% CI 1.06–1.99). Alternatively, CRS with fungal infection also had a higher significant association with overall autoimmune diseases (crude OR 2.49, 95% CI 2.14–2.91; adjusted OR 2.02, 95% CI 1.72–2.49), specifically for ankylosing spondylitis (adjusted OR 1.86, 95% CI 1.47–2.36) Behçet’s disease (adjusted OR 5.69, 95% CI 1.23–26.4), psoriasis (adjusted OR 2.05, 95% CI 1.54–2.74), rheumatoid arthritis (adjusted OR 1.69, 95% CI 1.08–2.65), sicca syndrome (adjusted OR 2.12, 95% CI 1.23–3.64), and systemic lupus erythematosus (adjusted OR 3.29, 95% CI 2.02–5.36). Table 7 demonstrates logistic regression for premorbid autoimmune diseases between CRS with fungal infection and non-fungal infection. CRS with fungal infection had a higher significant association with overall autoimmune diseases (adjusted OR 1.45, 95% CI 1.23–1.71) than CRS with non-fungal infection.

**Table 6:** Univariate and multivariate logistic regression for premorbid autoimmune diseases between CRS subgroups (fungal infection and non-fungal infection) and non-CRS group.

Variables	Non-fungal infection	Fungal infection	Odds ratio (non-fungal inf. vs. non-CRS)		Odds ratio (fungal inf. vs. non-CRS)	
	N = 26,778 n (%)	N = 3833 n (%)	Crude (95% CI)	Adjusted^a^ (95% CI)	Crude (95% CI)	Adjusted^a^ (95% CI)
Autoimmune diseases	813 (3.04)	182 (4.75)	1.56 (1.44–1.70)***	1.39 (1.28–1.51)***	2.49 (2.14–2.91)***	2.02 (1.72–2.49)***
Ankylosing spondylitis	391 (1.46)	77 (2.01)	1.62 (1.44–1.82)***	1.44 (1.28–1.62)***	2.24 (1.78–2.83)***	1.86 (1.47–2.36)***
Behçet’s disease	5 (0.02)	2 (0.05)	1.91 (0.67–5.41)	1.92 (0.66–5.57)	5.38 (1.21–23.9)*	5.69 (1.23–26.4)*
Dermatomyositis	1 (0.00)	0 (0.00)	0.51 (0.06–4.01)	0.60 (0.08–4.74)	–	–
Multiple sclerosis	0 (0.00)	0 (0.00)	–	–	–	–
Polymyositis	6 (0.02)	1 (0.03)	4.57 (1.48–14.2)**	3.45 (1.07–11.1)*	5.33 (0.64–44.3)	3.69 (0.43–31.9)
Psoriasis	172 (0.64)	52 (1.36)	1.21 (1.03–1.44)*	1.09 (0.92–1.29)	2.59 (1.95–3.44)***	2.05 (1.54–2.74)***
Rheumatoid arthritis	125 (0.47)	21 (0.55)	1.70 (1.39–2.09)***	1.59 (1.29–1.96)***	2.00 (1.29–3.12)**	1.69 (1.08–2.65)*
Sicca syndrome	90 (0.34)	15 (0.39)	2.66 (2.05–3.45)***	2.10 (1.61–2.75)***	3.10 (1.82–5.27)***	2.12 (1.23–3.64)**
SLE	55 (0.21)	19 (0.50)	1.71 (1.26–2.34)***	1.45 (1.06–1.99)*	4.15 (2.57–6.69)***	3.29 (2.02–5.36)***
Systemic sclerosis	5 (0.02)	0 (0.00)	0.79 (0.31–2.04)	0.74 (0.28–1.94)	–	–

*CI* confidence interval, *CRS* chronic rhinosinusitis, *SLE* systemic lupus erythematosus.

*p < 0.05, **p < 0.01, ***p < 0.001.

^a^Adjusted for gender, age, monthly income, urbanization, and comorbidities.

**Table 7:** Univariate and multivariate logistic regression for premorbid autoimmune diseases between CRS with fungal infection and with non-fungal infection.

Variables	Odds ratio (fungal inf. vs. non-fungal inf.)	
	Crude (95% CI)	Adjusted^a^ (95% CI)
Autoimmune diseases	1.59 (1.35–1.88)***	1.45 (1.23–1.71)***
Ankylosing spondylitis	1.38 (1.08–1.77)**	1.29 (1.01–1.65)*
Behçet’s disease	2.80 (0.54–14.4)	3.18 (0.62–16.5)
Dermatomyositis	–	–
Multiple sclerosis	–	–
Polymyositis	1.18 (0.14–9.70)	1.00 (0.12–8.41)
Psoriasis	2.13 (1.56–2.91)***	1.89 (1.38–2.60)***
Rheumatoid arthritis	1.18 (0.74–1.87)	1.05 (0.66–1.68)
Sicca syndrome	1.17 (0.67–2.02)	1.01 (0.58–1.76)
SLE	2.42 (1.44–4.08)***	2.23 (1.32–3.77)**
Systemic sclerosis	–	–

*CI* confidence interval, *CRS* chronic rhinosinusitis, *SLE* systemic lupus erythematosus.

*p < 0.05, **p < 0.01, ***p < 0.001.

^a^Adjusted for gender, age, monthly income, urbanization, and comorbidities.

## Discussion

This large-scale population-based study aimed to investigate the association between CRS and premorbid autoimmune diseases. In this study, autoimmune diseases were much more prevalent in patients with CRS than in persons without CRS (3.25% vs. 1.96%). Compared with persons without CRS, patients with CRS had a crude OR of 1.68 for premorbid autoimmune diseases (95% CI 1.56–1.81). The strength of the association between CRS and premorbid autoimmune diseases remained even after controlling for other potential risk factors (adjusted OR 1.39, 95% CI 1.28–1.50). Specifically, patients with CRS had a higher significant association with ankylosing spondylitis, polymyositis, psoriasis, rheumatoid arthritis, sicca syndrome, and systemic lupus erythematosus. When CRS was categorized into CRSsNP and CRSwNP, we found that premorbid autoimmune diseases accounted for 3.35% in patients with CRSsNP and 2.81% in patients with CRSwNP. In brief, CRSsNP and CRSwNP were both significantly associated with premorbid autoimmune diseases. In addition, when CRS was categorized into fungal and non-fungal infections, both subgroups were significantly associated with premorbid autoimmune diseases.

CRS has been recognized to be associated with comorbid medical conditions. Tan et al. conducted a case–control study and reported that patients with CRS have a significant association with adenotonsillitis, allergic rhinitis, otitis media, sleep apnea, asthma, gastroesophageal reflux disease, anxiety, and headache^18^. Chung et al. carried out a cross-sectional study, which revealed that patients with CRS exhibit a significant association with asthma, chronic pulmonary disease, obesity, migraine, lymphoma, depression, rheumatoid arthritis, peptic ulcer, cancer (solid tumor), tuberculosis, Parkinson’s disease, headache, hypothyroidism, cardiac arrhythmia, peripheral vascular disorder, stroke, psychosis, liver disease, hepatitis B/C, ankylosing spondylitis, hyperlipidemia, congestive heart failure, and hypertension^13^. In the present study, we found that patients with CRS had a higher significant association with autoimmune diseases, allergic rhinitis, asthma, chronic obstructive pulmonary disease, depression, gastroesophageal reflux disease, hyperlipidemia, ischemic heart disease, and obesity. These findings are compatible with the results of previous studies.

The possible mechanism of premorbid autoimmune diseases on the development of CRS is largely unknown. Genetic factors, immunity, and inflammatory processes may play essential roles in the association between premorbid autoimmune diseases and CRS. Alterations in mucociliary clearance, abnormalities in the sinonasal epithelial cell barrier, tissue remodeling, and innate and adaptive immune responses have been known to contribute to CRS pathogenesis^19^. It has been demonstrated that various cells such as fibroblasts, epithelial cells, endothelial cells, mast cells, eosinophils, antigen-presenting cells, T cells, and B cells coordinate the immune-inflammatory network underlying CRS by producing mediators, such as cytokines, chemokines, eicosanoids, and antibodies^20^. Underlying autoimmune diseases might influence CRS pathogenesis by altering these signaling pathways, inflammatory cytokines, and remodeling patterns^21,22^.

The highlight of the present study was to evaluate various autoimmune diseases in patients with CRS. Chandra et al. evaluated five autoimmune diseases and reported that the prevalence of CRS is relatively greater in patients with ankylosing spondylitis and rheumatoid arthritis than in those with lupus, psoriasis, and multiple sclerosis^12^. Chung et al. investigated three autoimmune diseases and reported that patients with CRS have a significant association with rheumatoid arthritis and ankylosing spondylitis but not with systemic lupus erythematosus^13^. This study evaluated 10 autoimmune diseases and found that patients with CRS were significantly associated with ankylosing spondylitis, polymyositis, psoriasis, rheumatoid arthritis, sicca syndrome, and systemic lupus erythematosus but not with Behçet’s disease, dermatomyositis, systemic sclerosis, and multiple sclerosis. The extremely low prevalence (< 0.05%) of Behçet’s disease, dermatomyositis, systemic sclerosis or absence of multiple sclerosis in our cohort has limited the evaluation of the association.

CRSwNP is an important clinical entity characterized by the presence of chronic sinonasal inflammation. Nasal polyps are inflammatory lesions that project into the nasal airway and originate from the ethmoid sinus. CRSwNP is frequently associated with allergic rhinitis and asthma, but the cellular and molecular mechanisms are not fully understood. Among all patients with CRS, approximately 25–30% have CRSwNP^4^. CRSsNP is more prevalent than CRSwNP, and its predisposing diseases include allergic and non-allergic airway diseases, immunodeficiencies, autoimmune diseases, and certain infectious diseases^5^. In the present study, CRSwNP accounted for 18.6% and CRSsNP accounted for 81.4% in patients with CRS. The prevalence of autoimmune diseases was 3.35% in CRSsNP and 2.81% in CRSwNP, both of which were significantly higher than the comparison persons (1.96%). Additionally, CRS can arise from chronic fungal infection and other conditions with different aspects of the immune system being activated. Our results showed that CRS with fungal infection and non-fungal infection accounted for 12.5% and 87.5% in patients with CRS, respectively. Furthermore, the prevalence of autoimmune diseases was 4.75% in CRS with fungal infection and 3.04% in CRS with non-fungal infection. Patients with fungal infection had a higher significant association to have overall autoimmune diseases than those with a non-fungal infection. Although fungal infection accounted for a higher proportion in CRSsNP (12.8%, n = 3180) than in CRSwNP (11.4%, n = 653; *p* = 0.006), both fungal and non-fungal infections did not influence the association between CRSwNP/CRSsNP and premorbid autoimmune diseases (data not shown).

The strength of our study included its use of population-based data to enroll sufficient CRS cases (n = 30,611) and controls (n = 122,444) to evaluate the occurrence of rare disorders (autoimmune diseases). Taiwan’s NHIRD is one of the largest nationwide population databases in the world, and no difference was found in the demographic distribution between LHID2000 and the original NHIRD. In addition, the universal coverage (> 99.9%) in the insurance program reduces barriers to health care access for all citizens, regardless of socioeconomic background and residential location^23^. By using the NHIRD, we were able to reflect a “real world” scenario in which CRS and all comorbidities were directly diagnosed during medical consultation. However, certain limitations to our findings should be considered. First, diagnoses were based on ICD format (for CRS, autoimmune diseases, and comorbidities), which is strongly dependent on the performance of physicians. However, audits are regularly performed to ensure that negligence and misdiagnoses are kept to a minimum. Second, the NHIRD does not contain detailed information on occupation, smoking habits, alcohol consumption, body mass index, diet preference, physical activity, environmental exposure, and family history, all of which are potential confounding factors. Several relevant clinical variables such as clinical data (cytokine data, inflammation markers, human leukocyte antigen type, etc.), imaging results, and culture reports were unavailable, which is a limitation of this study and should be addressed in future studies to obtain a better understanding of the meaning of the observed association between autoimmune disease and CRS.

## Conclusion

A significant association between CRS and premorbid autoimmune diseases has been identified. Specifically, patients with CRS had a higher significant association with ankylosing spondylitis, polymyositis, psoriasis, rheumatoid arthritis, sicca syndrome, and systemic lupus erythematosus. These underlying mechanisms need further investigation.

## Data Availability

The datasets analyzed in the current study can be accessed from the Taiwan National Health Insurance Research Database repository (https://nhird.nhri.org.tw/en).
